# A Device-Free Respiratory Gating Technique for Dynamic CT Using Time-Density Curve Analysis: A Technical Report With Clinical Applications

**DOI:** 10.7759/cureus.101427

**Published:** 2026-01-13

**Authors:** Nobuyuki Akiyama, Katsumi Tsujioka, Tomoya Ushiroda, Kazuyuki Ishihara

**Affiliations:** 1 Department of Radiology, Tosei General Hospital, Seto, JPN; 2 Department of Radiological Technology, Fujita Health University, Toyoake, JPN

**Keywords:** ct coronary angiography, device-free respiratory gating, dynamic computed tomography, four-dimensional computed tomography (4d-ct), time-density curve

## Abstract

Respiratory motion remains a major challenge in thoracic and cardiac CT. Conventional respiratory gating techniques often rely on external monitoring devices or dedicated software, which may complicate workflow and limit clinical availability. We developed a simple, device-free respiratory gating technique based on time-density curve (TDC) analysis derived directly from routinely acquired dynamic CT data. By placing a region of interest (ROI) on the chest wall, attenuation changes over time were analyzed to generate an intrinsic surrogate respiratory waveform, enabling retrospective identification of respiratory phases without additional hardware. The novelty of this approach lies in its use of intrinsic image data to generate a surrogate respiratory waveform, thereby eliminating the complexity and synchronization uncertainty associated with external monitoring devices.

The proposed workflow was implemented using standard dynamic CT protocols and applied to representative clinical applications, including lung tumor motion assessment for radiotherapy planning and free-breathing coronary CT angiography (FB-CCTA) in a patient unable to perform reliable breath-holding. This image-based approach provides a practical method for respiratory phase estimation and may expand the clinical applicability of dynamic CT imaging in situations where conventional respiratory control is impractical.

## Introduction

Respiratory motion plays different roles in CT depending on the clinical context. In radiotherapy planning, CT images are commonly acquired during free breathing, and respiratory-induced motion is intentionally captured and analyzed to estimate tumor excursion and define appropriate treatment margins [1,2]. In this setting, respiratory motion represents essential information rather than an artifact. In contrast, in diagnostic cardiac CT, particularly coronary CT angiography (CCTA), respiratory motion is an undesirable source of image degradation, and breath-holding is typically required to achieve adequate image quality [3,4].

To manage respiratory motion in radiotherapy, dedicated respiratory gating or tracking techniques have been widely adopted [5]. These approaches frequently rely on external monitoring devices, such as respiratory belts or infrared marker systems, to correlate internal anatomy with the respiratory cycle. Although effective, such systems increase procedural complexity and require additional hardware and workflow integration. Conversely, in diagnostic CT, external respiratory monitoring is uncommon, and respiratory control is generally achieved through patient breath-holding, which may be insufficient in patients unable to comply with breathing instructions.

Time-density curve (TDC) analysis has traditionally been used in CT imaging to determine optimal scan timing in contrast-enhanced studies [6]. When applied to time-resolved dynamic CT data, attenuation changes within a region of interest reflect underlying physiological motion. We hypothesized that TDC analysis derived from routinely acquired dynamic CT volumes could be repurposed to estimate respiratory phase by capturing chest wall motion directly from image data, thereby eliminating the need for external respiratory monitoring devices. The purpose of this technical report is to describe the workflow and implementation of a device-free respiratory gating technique based on TDC analysis and to demonstrate its feasibility through representative clinical applications in radiotherapy planning and diagnostic CT.

This work was previously presented in part as an abstract at the European Congress of Radiology (ECR) 2025, held between February 26 and March 2, 2025, in Vienna, Austria.

## Technical report

Technical description

Dynamic CT Acquisition

Dynamic volume CT scans were acquired using a wide-detector CT system capable of time-resolved volume imaging [7]. All examinations were performed under free-breathing conditions over a duration of 10 seconds. Because the normal adult respiratory rate is approximately 12-20 breaths per minute [8], this duration ensured that at least one full respiratory cycle was captured. Scan parameters were selected according to institutional protocols and specific clinical applications. For cardiac imaging applications, ECG gating was applied concurrently. No external respiratory monitoring devices or dedicated respiratory gating software were used.

ROI Placement and TDC Generation

Respiratory motion was estimated using TDC analysis derived directly from dynamic CT data. A circular ROI was retrospectively placed on the reconstructed dynamic CT images using the vendor-provided TDC analysis function integrated into the Aquilion ONE (Canon Medical Systems, Otawara, Tochigi, JPN) CT system. The ROI was positioned within the chest wall region in an area with sufficient soft-tissue contrast, avoiding bony structures and lung parenchyma, and with minimal influence from cardiac motion. The typical ROI diameter was approximately 20-22 mm, depending on the patient's body habitus and image resolution.

**Figure 1:**
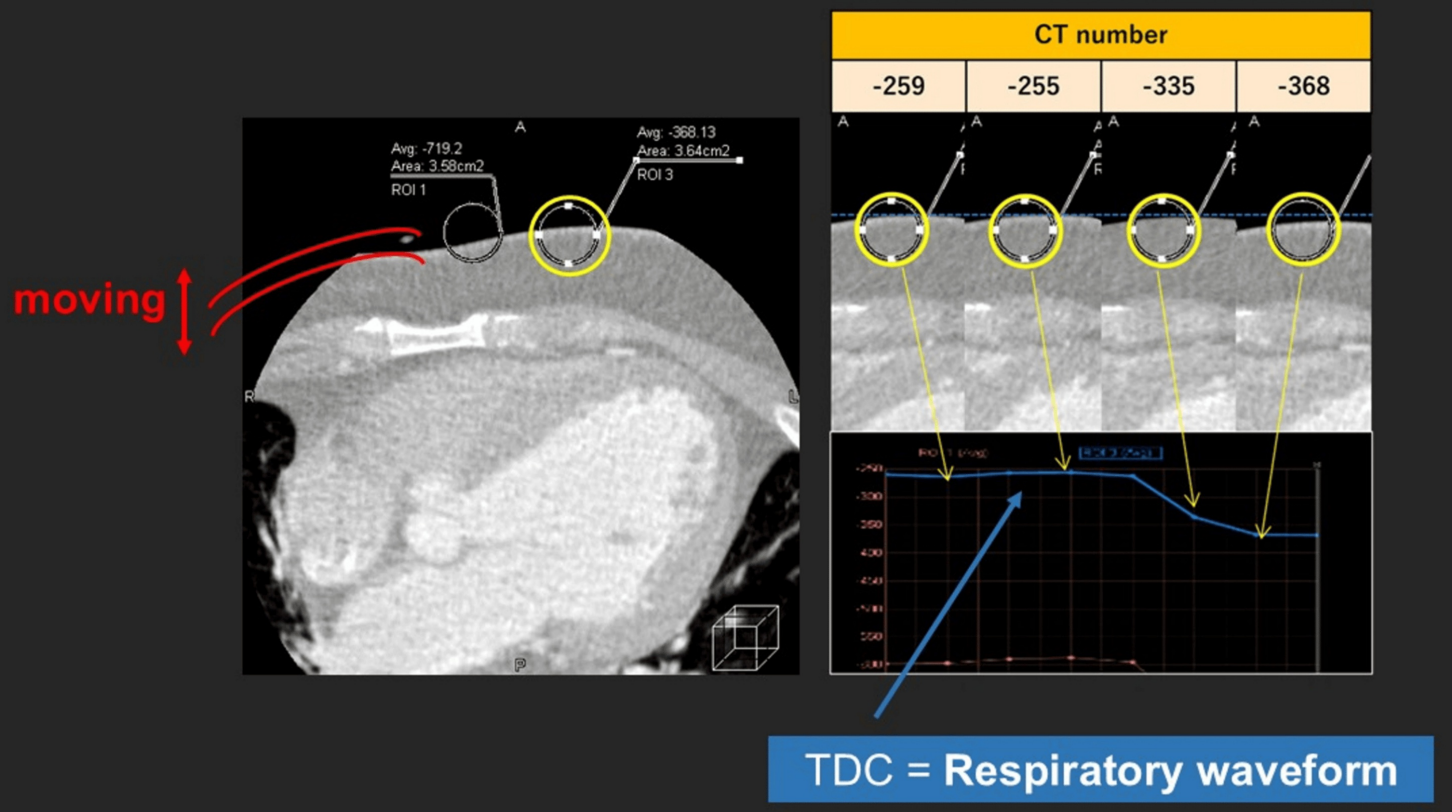
Workflow for device-free respiratory phase estimation using TDC analysis An ROI is placed on the chest wall within time-resolved dynamic CT volume data. Attenuation changes within the ROI are plotted over time to generate a TDC, which serves as an intrinsic surrogate respiratory waveform for retrospective identification of respiratory phases. ROI: Region of interest, TDC: Time-density curve

The ROI was not intended to measure absolute attenuation values or tissue composition. Instead, relative temporal changes in attenuation within the ROI were analyzed to generate a TDC, allowing respiratory phase estimation based on waveform periodicity rather than predefined density thresholds. An overview of the proposed device-free respiratory phase estimation workflow is illustrated in Figure 1.

Respiratory Phase Identification

Respiratory phases were identified based on waveform characteristics of the TDC. Local maxima and minima were used as reference points for defining respiratory states, while intervals with minimal attenuation change were interpreted as respiratory pause phases. These time points or intervals were subsequently used for respiratory phase-guided image reconstruction or post-processing.

Image Reconstruction and Workflow Summary

**Figure 2:**
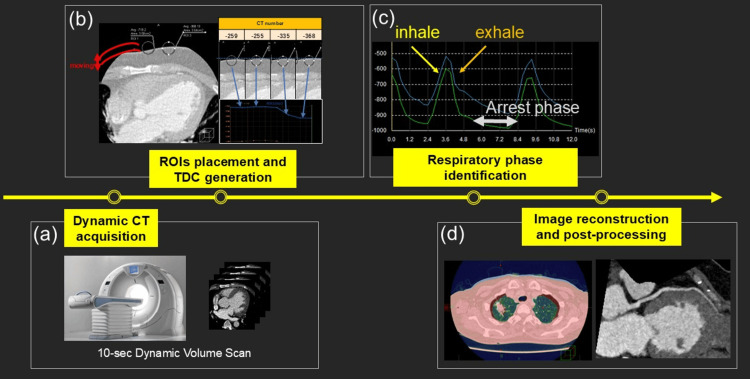
Schematic overview of the proposed device-free respiratory gating workflow

Image reconstruction was performed using standard reconstruction algorithms available on the CT system. When appropriate, advanced iterative reconstruction techniques were applied to improve image quality under dynamic or low-dose conditions [9]. The proposed workflow consists of four steps: (1) acquisition of free-breathing dynamic CT data, (2) ROI placement and TDC generation, (3) respiratory phase identification, and (4) respiratory phase-guided reconstruction or analysis. A schematic summary of the workflow is shown in Figure 2. Representative CT acquisition parameters and application settings for each clinical application are summarized in Table 1.

**Table 1:** Representative CT acquisition parameters and application settings for the proposed device-free respiratory gating technique FB-CCTA: Free-breathing coronary CT angiography

Parameter	Application 1: Radiotherapy planning (lung tumor motion assessment)	Application 2: FB-CCTA
CT system	Aquilion ONE	Aquilion ONE
Scan mode	Dynamic volume scan	ECG-gated dynamic volume scan
Breathing condition	Free breathing	Free breathing
Scan duration	10 s	10 s
Tube voltage	120 kVp	120 kVp
Tube current	50 mA	150 mA
Rotation time	0.5 s/rotation	0.275 s/rotation
Detector collimation	320 × 0.5 mm	320 × 0.5 mm
ECG gating	Not applied	Prospective ECG gating
Target cardiac phase	–	30–R%
Contrast agent	Not used	Iodinated contrast medium
Contrast volume	–	75 mL
Injection rate	–	5.0 mL/s

Clinical applications

Application 1: Lung Tumor Motion Assessment for Radiotherapy Planning

**Figure 3:**
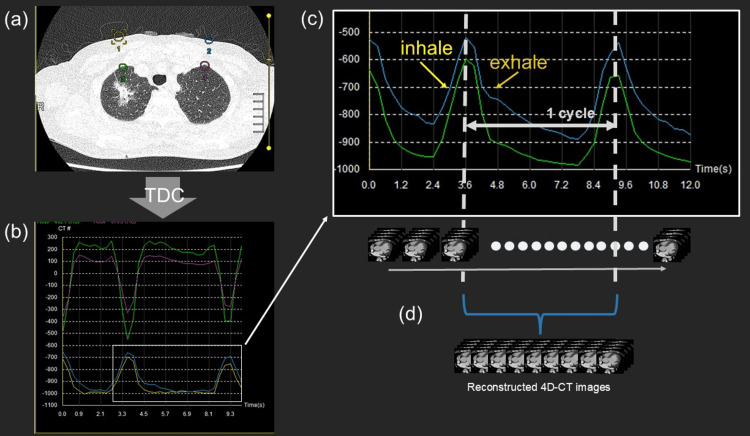
Respiratory phase-resolved image reconstruction for lung tumor motion assessment (a) Placement of ROIs on the chest wall within time-series dynamic CT data (b and c) Representative TDCs reflecting respiratory motion are used for respiratory phase identification (d) Image volumes reconstructed at multiple time points within a single respiratory cycle to visualize respiratory-induced tumor motion ROI: Region of interest, TDC: Time-density curve

**Figure 4:**
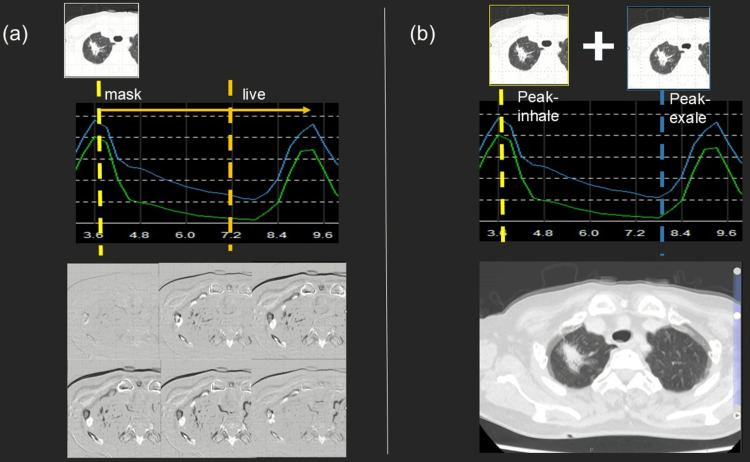
Visualization of lung tumor motion using image-processing techniques (a) Subtraction imaging using the peak inspiration volume as a mask, analogous to digital subtraction angiography, enabling visualization of tumor displacement throughout the respiratory cycle. (b) Additive imaging combining peak inspiration and peak expiration volumes to highlight the overall extent of tumor motion.

The proposed technique was applied to free-breathing dynamic CT data acquired for lung tumor motion assessment. Respiratory phases were retrospectively identified using TDC analysis, and image volumes corresponding to different points within a single respiratory cycle were reconstructed. Respiratory phase-resolved image reconstruction is shown in Figure 3. Visualization of tumor motion using subtraction and additive image-processing techniques is demonstrated in Figure 4. This application demonstrates that respiratory phase information derived from dynamic CT data can support tumor motion assessment without external respiratory monitoring.

Application 2: Free-Breathing Coronary CT Angiography

**Figure 5:**
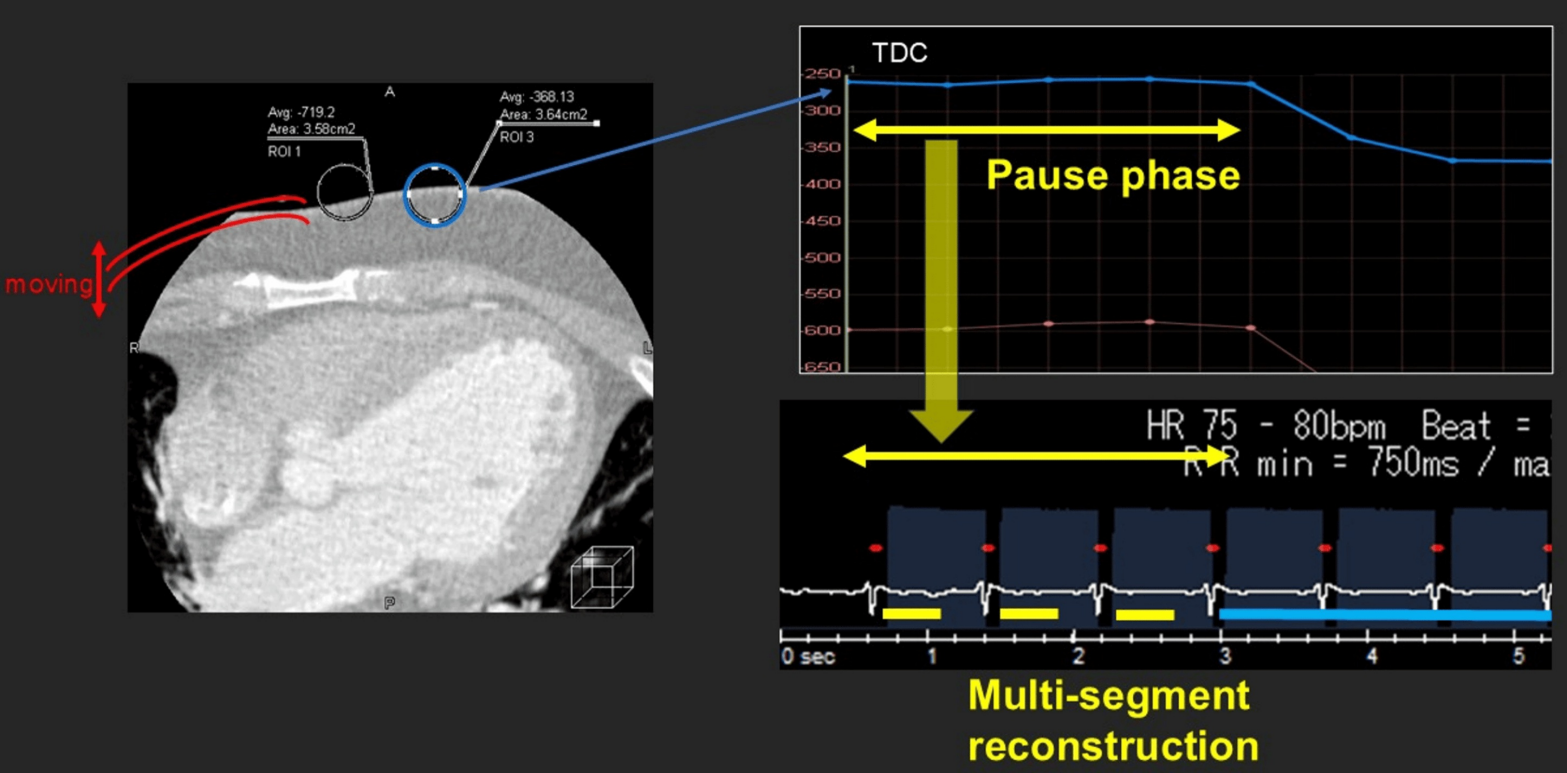
Identification of respiratory pause phases for FB-CCTA Respiratory pause phases were retrospectively identified using a TDC generated from a chest wall ROI. Time intervals corresponding to minimal respiratory motion were selected for subsequent respiratory phase–selective image reconstruction. FB-CCTA: Free-breathing coronary CT angiography, TDC: Time-density curve, ROI: Region of interest, HR: Heart rate, RR: Respiratory rate

**Figure 6:**
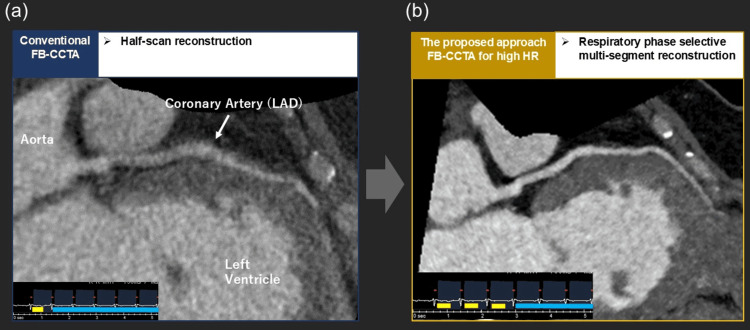
Comparison of coronary image quality between conventional and respiratory phase-selective reconstruction Representative coronary CT angiography images reconstructed using (a) conventional half-scan reconstruction under free-breathing conditions and (b) respiratory phase-selective multi-segment reconstruction based on TDC-derived respiratory phase information. Respiratory phase–selective reconstruction demonstrates reduced motion artifacts and improved coronary delineation. FB-CCTA: Free-breathing coronary CT angiography, TDC: Time-density curve, HR: Heart rate, LAD: Left anterior descending artery

Respiratory and cardiac motion can substantially degrade coronary image quality, particularly under free-breathing conditions and elevated heart rates [4,10,11]. In this application, respiratory pause phases were retrospectively identified using TDC analysis of chest wall motion. Multi-segment reconstruction was performed using projection data acquired within the identified respiratory pause phase, based on established principles for high heart rate imaging [3]. Identification of respiratory pause phases is illustrated in Figure 5, and a comparison of conventional and respiratory phase-selective reconstruction is shown in Figure 6.

## Discussion

Conventional respiratory gating techniques typically depend on external surrogate signals, which may increase workflow complexity and introduce synchronization uncertainty between external markers and internal organ motion [5,12,13]. In contrast, the proposed approach derives respiratory phase information directly from routinely acquired dynamic CT data.

Using chest wall motion as an intrinsic surrogate aligns with prior image-based and data-driven respiratory gating concepts reported in other imaging modalities [14]. The clinical applications presented in this report demonstrate that a simple TDC derived from dynamic CT images can provide sufficient respiratory phase information to support both therapeutic and diagnostic imaging workflows. Importantly, the same underlying principle was applicable across different clinical contexts, including lung tumor motion assessment and FB-CCTA, highlighting the versatility of the proposed method.

This report focuses on technical feasibility and workflow description rather than quantitative performance evaluation. Because the ROI was placed within the chest wall region, partial volume effects at the chest wall-lung interface may contribute to attenuation changes across respiratory phases. However, this variability does not adversely affect respiratory phase identification, as the proposed method relies on relative temporal changes in attenuation rather than absolute CT numbers or tissue composition. The periodic waveform characteristics of the TDC, rather than predefined density thresholds, form the basis for respiratory phase estimation. Furthermore, attenuation characteristics may vary depending on beam geometry, reconstruction algorithms, or system-specific calibration. Nevertheless, because the proposed approach is based on relative temporal attenuation changes within a fixed image-based ROI, differences in absolute attenuation values are not expected to compromise respiratory phase identification within the scope of diagnostic CT imaging addressed in this study.

Another limitation of this work is the small number of representative clinical applications and the absence of direct comparison with device-based respiratory gating systems. Consequently, conclusions regarding broad clinical effectiveness or superiority over established techniques cannot be drawn at this stage. Future studies involving larger patient cohorts and quantitative image quality assessment will be required to further validate the clinical utility of this approach.

Radiation dose is an important consideration in both routine CT imaging and dynamic CT workflows. Deep learning-based reconstruction techniques have been shown to enable considerable dose reduction while maintaining diagnostic image quality by reducing image noise and improving contrast-to-noise ratios compared with traditional reconstruction methods [15]. Additionally, emerging detector technologies such as photon-counting CT (PCCT) hold promise for further dose efficiency improvements by enhancing spatial resolution and reducing electronic noise relative to conventional detectors [16]. These advancements suggest that future integration of advanced reconstruction and detector technologies with image-based respiratory gating may improve both image quality and patient safety.

## Conclusions

This study proposes a device-free respiratory gating technique based on TDC analysis of dynamic CT data and demonstrates its technical feasibility. By utilizing attenuation changes in a chest wall ROI as an intrinsic respiratory surrogate, respiratory phase information can be retrospectively estimated without external monitoring devices. This approach enables respiratory phase-resolved or phase-selective image reconstruction using routinely acquired CT data and may expand the clinical applicability of dynamic CT imaging when conventional respiratory control methods are impractical.
